# Computed-Tomography-Based Radiomics Model for Predicting the Malignant Potential of Gastrointestinal Stromal Tumors Preoperatively: A Multi-Classifier and Multicenter Study

**DOI:** 10.3389/fonc.2021.582847

**Published:** 2021-04-22

**Authors:** Minhong Wang, Zhan Feng, Lixiang Zhou, Liang Zhang, Xiaojun Hao, Jian Zhai

**Affiliations:** ^1^Department of Radiology, The First Affiliated Hospital of Wannan Medical College, Wuhu, China; ^2^Department of Radiology, College of Medicine, The First Affiliated Hospital, Zhejiang University, Hangzhou, China; ^3^Department of Pharmacy, The First Affiliated Hospital of Wannan Medical College, Wuhu, China; ^4^Department of Radiology, Zhejiang Cancer Hospital, Hangzhou, China

**Keywords:** gastrointestinal stromal tumor, risk classification, radiomics, X-ray computer, multi-classifier

## Abstract

**Background:** Our goal was to establish and verify a radiomics risk grading model for gastrointestinal stromal tumors (GISTs) and to identify the optimal algorithm for risk stratification.

**Methods:** We conducted a retrospective analysis of 324 patients with GISTs, the presence of which was confirmed by surgical pathology. Patients were treated at three different hospitals. A training cohort of 180 patients was collected from the largest center, while an external validation cohort of 144 patients was collected from the other two centers. To extract radiomics features, regions of interest (ROIs) were outlined layer by layer along the edge of the tumor contour on CT images of the arterial and portal venous phases. The dimensionality of radiomic features was reduced, and the top 10 features with importance value above 5 were selected before modeling. The training cohort used three classifiers [logistic regression, support vector machine (SVM), and random forest] to establish three GIST risk stratification prediction models. The receiver operating characteristic curve (ROC) was used to compare model performance, which was validated by external data.

**Results:** In the training cohort, the average area under the curve (AUC) was 0.84 ± 0.07 of the logistic regression, 0.88 ± 0.06 of the random forest, and 0.81 ± 0.08 of the SVM. In the external validation cohort, the AUC was 0.85 of the logistic regression, 0.90 of the random forest, and 0.80 of the SVM. The random forest model performed the best in both the training and the external validation cohorts and could be generalized.

**Conclusion:** Based on CT radiomics, there are multiple machine-learning models that can predict the risk of GISTs. Among them, the random forest algorithm had the highest prediction efficiency and could be readily generalizable. Through external validation data, we assume that the random forest model may be used as an effective tool to guide preoperative clinical decision-making.

## Introduction

Gastrointestinal stromal tumors (GISTs) are the most common mesenchymal tumors of the digestive system, which occur in the stomach and small intestine. GISTs have a variety of biological characteristics and cannot be simply categorized as benign or malignant (1). For example, some small GISTs can progress rapidly and metastasize to the liver, while some large GISTs, even those not receiving the post-operative adjuvant treatment, present no long-term risk of recurrence or metastasis (2). Therefore, the preoperative evaluation of the malignant potential of GISTs is crucial for treatment decision-making.

Risk stratification is commonly applied to evaluate the biological behaviors and overall clinical outcome of GISTs. Currently, the most recognized criterion is the improved National Institutes of Health risk stratification standard introduced by Joensuu in 2008 (3), which is based on tumor maximum diameter and mitotic count and introduces two parameters: tumor site and tumor rupture. The risk of relapse is thereby divided into four categories: very low risk, low risk, intermediate risk, and high risk. Higher risk generally indicates a worse prognosis. Also, the introduction of imatinib mesylate has greatly changed the outcomes in high-risk GIST patients (4). The need for reliable preoperative risk stratification is of great significance for the development of treatment methods and prognostic evaluation. Most surgeries can completely remove the GISTs without first conducting a preoperative biopsy (5), which may cause tumor ulceration and bleeding, increasing the risk of tumor spread. Therefore, it is of great clinical value to explore non-invasive, reliable, and simple biomarkers for predicting the recurrence and metastasis risk of GISTs before surgery.

Previous GIST risk stratification research is largely based on analysis of computed tomography (CT) images (4, 6–9), which is likely influenced by the observer's subjective assessment. Therefore, an objective and quantitative technique is urgently needed for the accurate risk stratification of GISTs. Radiomics converts medical images into high-dimensional data that can be mined, which holds great potential for application in disease diagnosis, identification, and prognosis predictions (10–13).There are studies have examined the utility of radiomics in GIST risk stratification (14–16) and have achieved favorable results. However, most of these studies are single-center trials, whose prediction models have not been externally verified. Therefore, the generalizability of these models remains unclear. In addition, previous studies used a single classifier for modeling, due to the obvious differences in classifier algorithms (17), and such studies are unable to determine the classifier with the best performance in risk prediction.

In response to these shortcomings, we conducted a multiclassifier and multicenter GISTs radiomics study, applying the three most commonly used machine-learning classifiers in radiomics to the same cohort of data to evaluate and compare the performance of the classifiers. Also, the model was tested with independent external data to further evaluate its generalizability to provide a reference for clinical treatment decisions.

## Materials and Methods

### Patients

Data from a total of 324 patients with GISTs presenting from January 1, 2016 to July 1, 2019 were collected retrospectively from three hospitals. Among them, 180 cases were analyzed from the First Affiliated Hospital of Zhejiang University School of Medicine, which was used as the training cohort, while 144 cases from another two hospitals (Zhejiang Cancer Hospital and the First Affiliated Hospital of Wannan Medical College) were used as the external validation cohort. The inclusion criteria were as follows: (1) surgical resection, negative margin, and a pathological diagnosis of GISTs, (2) abdominal enhancement CT examination within 15 days prior to surgery, and (3) pathological results with a clear risk assessment. Exclusion criteria were as follows: (1) patients receiving imatinib or other neoadjuvant therapy before surgery, and (2) those with poor CT image quality.

Clinical data, including age, gender, and tumor site, were derived from medical records. The National Institutes of Health's modified criteria were used to stratify the malignant potential of GISTs on the basis of the clinical and post-operative histological index. All patients were divided into two groups: high malignant potential group with intermediate risk and high risk; and low malignant potential group with very low risk and low risk. This study was a retrospective study, and the patient's informed consent was thereby waived, as approved by the hospital ethics committee.

### CT Image Acquisition

All subjects received a default abdominal CT scan using one of the three multidetector CT (MDCT) systems with the scanning and reconstruction parameters used in daily clinical practice. See Table 1 for the detailed information of the CT protocol. Three-phase scans were unenhanced phase, arterial phase (25–30 s after injection), and portal vein phase (55–60 s after injection). The dose of iodine contrast agent was based on the patient's weight (1 mL/kg), and the flow rate was 2.5–3.5 mL/s.

**Table 1:** The protocols of the CT scan for the patients with GISTs.

**Manufacture**	**Philips**	**SIEMENS**	**Philips**
CT scanner	Brilliance 64	Dual source CT	Brilliance 256
Tube voltage (kV)	120	120	120
Tube current (mA)	250	200	250
Rotation time (s)	0.4	0.5	0.5
Detector collimation (mm)	64 × 0.625	128 × 0.6	64 × 0.625
Pitch	0.891	0.6	0.914
Slice thickness (mm)	5	5	5
Slice spacing (mm)	5	5	5
Matrix	512 × 512	512 × 512	512 × 512
FOV (mm)	350	300	350
Algorithm (B)	Standard	Standard	Standard

### Three-Dimensional Segmentation of Tumor Images and Radiomics Feature Extraction

Both tumor segmentation and radiomics feature extraction were performed using Matlab's IBEX software package (18). Two radiologists with a depth of experience delineated the regions of interest (ROIs) layer by layer along the edge of the tumor contour on the CT images of the arterial and portal venous phases.

All images were preprocessed with image resampling (voxel size of 1 × 1 × 1 mm^3^) and gray value homogenization (normalized to 1–256, fixed bin number method, 256 bins) before radiomics feature extraction. The radiological feature parameters involved six major categories: histogram parameters (*n* = 48), 2.5D and 3D gray level co-occurence matrix (*n* = 594, the 2.5D feature is computed from a single matrix after merging all 2D directional matrices, the 3D feature is computed from a single matrix after merging all 3D directional), gray level adjacent difference (*n* = 10), gray level run length matrix (*n* = 34), shape and size (*n* = 18). In each stage, we retrieved 704 parameters, and a total of 1,408 parameters were collected in the two stages.

During the early stage of the study, we randomly selected images from 40 patients, and two radiologists with more than 10 years of work experience performed ROI delineation independently. The blindness method was used to analyze the reliability and repeatability between observers. The consistency was evaluated using the intra-class correlation coefficient (ICC). There is a good agreement when the ICC is > 0.75. ROI extraction of the remaining images was performed by one of the radiologists.

### Feature Selection and Radiomics Model Building

Redundancy and overcorrelation in the characteristics of radiomics often lead to overfitting of the prediction model. In this study, we dimensionally reduced the radiomics features in two steps. First, multicollinearity of the features were analyzed by spearman correlation, and the correlation coefficient threshold was 0.8. Then, we used the boruta algorithm to iteratively assess the importance of features, and we removed the irrelevant features. Boruta algorithm can filter out all the characteristics related to the dependent variable and generate a ranking of importance. To achieve statistical significance, the top 10 features in importance ranking were selected for final modeling.

After dimensionality reduction of the radiomics features, the three most popular classifiers [logistic regression, support vector machine (SVM), and random forest] were applied to establish three risk stratification models for radiological prediction. We conducted holdout cross-validation for 30 times for each model in the training cohort (training: internal validation ratio is 4:1). Because each iteration is a resampling of the training cohort, each model yielded 30 different values of area under the curve (AUC), specificity, sensitivity, and accuracy, among which we used AUC as the standard to evaluate the effectiveness of the three models in the training cohort.

Subsequently, the three models were applied to the external validation cohort, and the effectiveness of the models were also evaluated through AUC, specificity, sensitivity, and accuracy.

### Statistical Analysis

All statistical analysis was performed using R software (version 3.4.1; http://www.Rproject.org). We performed descriptive statistical analysis for the training and external validation cohorts, and quantitative data was described as mean ± standard deviation (SD) and qualitative data was described by frequency (percent). Qualitative variables were compared using the chi-square test. Continuous variable data was evaluated using a two-sample *t-*test or Wilcoxon test. AUC was used as the evaluation standard for the comparison of the three classification algorithms in the training cohort. The Fridman test was used for the comparison among the three algorithms, and the Nemenyi test was used in *post-hoc* analysis. Two tailed *p* < 0.05 was considered statistically significant.

## Results

### Clinical Characteristics

In total, 324 GIST patients were included in this study, of which 150 patients had low malignant potential and 174 patients had high malignant potential. Ninety-three men and 87 women were included in the training cohort, and 64 men and 80 women were included in the external validation cohort. Table 2 shows the baseline clinical data. Single factor analysis showed that there was no statistically significant difference between the low and the high malignant potential groups in terms of age, gender, and tumor site.

**Table 2:** Patient characteristics in the training and external validation cohorts.

**Patient characteristics**	**Training cohort**			**External validation cohort**		
	**Low-malignant potential GISTs (*n =* 82)**	**High-malignant potential GISTs (*n =* 98)**	***p*-value**	**Low-malignant potential GISTs (*n =* 68)**	**High-malignant potential GISTs *n =* 76)**	***p*-value**
Age (mean ± SD, years)	54.13 ± 8.31	56.71 ± 10.52	0.74	55.13 ± 8.31	57.12 ± 11.45	0.63
Gender (%)			0.15			0.77
Male	37 (45.12%)	56 (57.14%)		31 (45.59%)	33 (43.42%)	
Female	45 (54.88%)	42 (42.86%)		37 (54.41%)	43 (56.58%)	
Primary site (%)			0.65			0.19
Gastric	48 (58.53%)	53 (54.08%)		45 (66.18%)	42 (55.26%)	
Intestinal	34 (41.47%)	45 (45.92%)		23 (33.82%)	34 (44.74%)	

After dimension reduction by spearman correlation, we obtained 107 features, which through the dimension reduction by boruta algorithm, 25 parameters remained, from which we extracted the top 10 features, according to the built-in importance-ranking system. In the subset, parameters from the portal venous phase accounted for 80%. Morphology ranks the most important, although only one parameter was selected. See Table 3 for a list of specific parameters and their importance.

**Table 3:** Texture features selection for radiomics models.

**Parameters category**	**Parameters**	**Phase**	**Importance**
Morphology	Volume	Portal venous phase	21.11
Gray level co-occurrence matrix	Variance	Portal venous phase	9.26
Gray level co-occurrence matrix	Inverse variance	Arterial phase	8.04
Gray level co-occurrence matrix	Cluster shade	Portal venous phase	7.78
Gray level adjacent difference	Contrast	Portal venous phase	7.59
Gray level co-occurrence matrix	Max probability	Arterial phase	6.17
Gray level adjacent difference	Busyness	Portal venous phase	5.39
Gray level co-occurrence matrix	Sum average	Portal venous phase	5.23
Gray level adjacent difference	Texture strength	Portal venous phase	5.15
Gray level adjacent difference	Complexity	Portal venous phase	5.14

### Radiomics Model Performance

**Figure 1:**
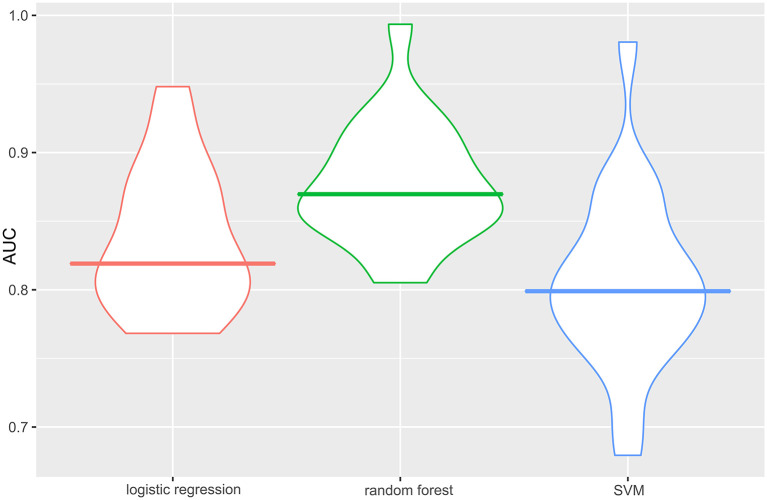
AUC of the three classifier prediction models performance in the training cohort. The random forest model achieved the best satisfactory results. The AUC is the average AUC obtained after 30 holdout cross-validation. The horizontal line of each diagram corresponds to the average AUC. AUC, the area under the curve; SVM, support vector machine.

**Figure 2:**
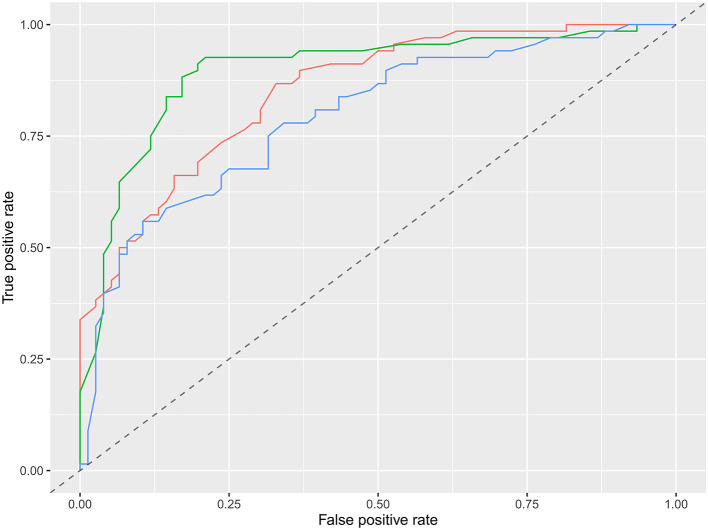
ROC diagram of multiple models in the external validation cohort. Red is logistic regression, green is random forest, and blue is support vector machine.

The specific performance of the three classifier prediction models is shown in Table 4 and Figures 1, 2. The Friedman test indicated that the AUC value of the three models in the training cohort was significantly different (*p* < 0.001). The Nemenyi test results show that the AUC of random forest was significantly higher than logistic regression (*p* = 0.001), significantly higher than SVM (*p* = 0.0103), and there was no significant statistics between logisitic regression and SVM (*p* = 0.09). The Friedman-Nemenyi test indicated that the AUC value of the random forest model was significantly higher than that of the other two prediction models. The random forest model achieved the most satisfactory results; the performance and generalizability were favorable. The performance of the SVM and logistic regression models were satisfactory, and the generalizability was acceptable, but the overall efficiency was not outstanding.

**Table 4:** A performance summary of the radiomics models in the training and external validation cohorts.

	**Accuracy**	**Sensitivity**	**Specificity**	**AUC**
Logistic regression				
Training cohort	0.77 ± 0.08	0.61 ± 0.11	0.86 ± 0.10	0.84 ± 0.07
External validation cohort	0.75	0.65	0.84	0.85
Random forest				
Training cohort	0.82 ± 0.07	0.84 ± 0.10	0.73 ± 0.10	0.88 ± 0.06
External validation cohort	0.84	0.93	0.76	0.90
Support vector machine				
Training cohort	0.75 ± 0.07	0.52 ± 0.12	0.91 ± 0.08	0.81 ± 0.08
External validation cohort	0.71	0.74	0.68	0.80

*Values of accuracy, sensitivity, specificity, and AUC of the three models in the training cohort are the average values after 30 holdout cross-validation, which were described as mean ± standard deviation (SD). AUC, areas under the curve*.

## Discussion

In this study, we built three prediction models based on CT radiomics for GIST risk stratification. After comparing the three most commonly used machine-learning models in radiomics, we found the random forest model showed the best performance in discriminating GISTs malignant potentials, and its generalizability is outstanding.

GISTs often exhibit complex and unpredictable biological behaviors. With the development of molecular pathology research, imatinib has emerged as a first-line molecular targeted drug, which has changed the treatment of GISTs and has become a successful model for the targeted diagnosis and treatment of solid tumors. The stratification of patients based on the risk of recurrence is a key issue in managing primary GISTs. The National Comprehensive Cancer Network guidelines recommend more than 3 years of post-operative imatinib be used as an adjuvant therapy for patients with a high recurrence risk (high-risk and intermediate-risk) (19, 20), while patients with a low recurrence risk (low-risk and very low-risk) that can be cured *via* surgical resection of the tumor should not receive adjuvant therapy with imatinib (21–23). Therefore, in this study, GIST patients were classified into low and high malignant potential groups according to the risk stratification. Because the clinical characteristics of GISTs lack specificity, the preoperative diagnosis and risk stratification of GISTs mainly rely on imaging examinations. Traditional imaging evaluates the risk of GISTs by observing the size, shape, presence or absence of necrosis, ulcers, and enhancement of GISTs, and the results depend much on the professional ability and subjective experience of radiologists (4, 6–9).

The rise in the use of radiomics in recent years has resulted in imaging studies to predict GISTs recurrence risks using objective and quantitative measures. Currently, most GISTs radiomics studies focus on risk prediction, and the AUC is relatively high at ~0.81–0.94 (15, 19, 24–27), demonstrating the superiority of radiomics over traditional methods in terms of prediction effectiveness. It also lays foundation for the future application of radiomics for GIST risk stratification. However, only one study has also conducted external data validation of the model (24). Its model efficiency was 0.87 in the training cohort and 0.85 in the external validation cohort. Although the performance of the model was not optimal, this study has published the most standardized and reliable results to date. There is no external validation for the other studies; the same data were used for the training and validation cohort, making the results less convincing (28). Studies have confirmed that equipment from different manufacturers results in differences in scanning parameter settings and post-processing reconstruction algorithms, resulting in significant differences in the radiomics parameters (29–31). Therefore, single-center research has its limitations (32). Multicenter research can provide diverse imaging data to better interpret tumor heterogeneity, which is also in line with the development of precision medicine (33). The highlight of this research lies in its multicenter design, which uses the largest amount of data among the three hospitals as the training cohort, while the data from the other two hospitals are fused into an independent external validation cohort. We found that the AUC of the random forest model in the training cohort was 0.88 ± 0.06, which was very good in both the training cohort and the validation cohort, indicating that the generalizability of the model is excellent. Our study confirms the potential of radiomics in GISTs diagnosis and prognosis, and it proposes that the predicted models must undergo multicenter testing before providing a reliable reference for clinical decision-making (34).

Different machine-learning algorithms have their own advantages and disadvantages. The performance of an algorithm in a specific machine-learning task cannot be predicted before research. Most previous radiomics studies used a single algorithm for modeling, and no specific reason was stated for choosing the model. Currently, the most common GIST risk stratification models are logistic regression, SVM, and random forest. Logistic regression is the most commonly used classification algorithm in the medical field (35) and in GISTs imaging histology. Wang et al. (26). collected 333 GISTs cases, and the AUC of the training cohort was 0.88. Ren et al. (27) also used logistic regression with 440 cases, and the final AUC of the training cohort was 0.93. SVM has many advantages in processing small samples and non-linear and high-dimensional data. Chen et al. chose SVM to build a prediction model, and the AUC was 0.86 in the training cohort and 0.85 in the external validation cohort. Random forest is a type of integrated machine learning, which is based on the decision tree method and can improve the prediction accuracy without significantly increasing the amount of calculation (36). Zhang et al. (19) used a random forest algorithm to predict GIST risk stratification, and achieved an AUC of 0.94 of the training cohort, which is the best performance among similar studies. These studies have their own advantages, but due to the heterogeneity between the data cohorts, the differences of the classifiers cannot be clarified. Hence, it is impossible to determine which classifier is the most suitable for stratifying the GIST risk. In this study, we conducted a multiclassification algorithm study on the same data and task and found that logistic regression and SVM performed stably, but the overall efficiency was not outstanding. Random forest performed the best in both the training and external validation cohorts, with the highest AUC and excellent generalizability, which indicated that this method is worthy of in-depth study and verification with a larger sample set and data from a multicenter study.

However, our study has the following limitations: (1) Our sample size was relatively small, and limited to Chinese people. As genetic mutations are the driving factors in the occurrence of GISTs, and the morbidity and mortality of GISTs varies among different races, it is necessary to conduct further in-depth studies on large samples of multinational and multiethnic populations, ideally in multicenter trials. (2) Because most of the previous articles suggested clinical parameters were not significant, this study used pure radiomic modeling and did not integrate clinical parameters for further analysis and comparison. (3) This study was a retrospective study, and the sample selection was biased, which requires further verification in prospective studies. (4) As the CT imaging protocols varies in different hospitals, radiomics features are affected by CT scanner parameters, such as reconstruction kernel or section thickness, thus obscuring underlying biologically important radiomics parameters. We did not process the data from multicenter with harmonization. Some features of IBEX are not compatible with IBSI (Image Biomarker Standardisation Initiative), which will affect the reproducibility of the results. (5) The algorithm of feature selection also affects the model performance. We did not compare the algorithms of dimensionality reduction; therefore, the final feature selection may not be the optimal.

In conclusion, this study predicts the risks of GISTs based on different machine-learning models of CT radiomics. After comparing the three most commonly used machine-learning algorithms in radiomics, a radiomics model of the random forest algorithm presents the most satisfactory prediction. The efficacy, optimal discrimination, strong generalizability, and confirmation in external validation data can be used as a more objective and non-invasive technique, which has the potential to become an effective tool for clinicians to predict the risk stratification of GISTs before surgery.

## Data Availability Statement

The raw data supporting the conclusions of this article will be made available by the authors, without undue reservation.

## Ethics Statement

The studies involving human participants were reviewed and approved by Institutional review board of the Affiliated Hospital of College of Medicine Zhejiang University. Written informed consent for participation was not required for this study in accordance with the national legislation and the institutional requirements.

## Author Contributions

MW and ZF proposed the conception and design of this research and analyzed and interpreted the data. ZF and LZho developed methodology. MW, ZF, LZha, and XH collected data and performed preprocessing. MW, ZF, and JZ were major contributors in writing the manuscript. All authors read and approved the final manuscript.

## Conflict of Interest

The authors declare that the research was conducted in the absence of any commercial or financial relationships that could be construed as a potential conflict of interest.
